# Impact of artificial feeding policies on space use and competition dynamics in overwintering hooded crane and goose populations

**DOI:** 10.1371/journal.pone.0336710

**Published:** 2025-11-12

**Authors:** Jong-Ju Son, Ju-Hyun Lee, Jung-Moon Ha, Na-Ru Kang, Sun-Mee Hwang, Jae-Ung Jang, Dae-Han Cho, Se-Yeong Kim, Won-Suk Choi, Yong-Un Shin, Ha-Cheol Sung

**Affiliations:** 1 School of Biological Sciences and Biotechnology, Chonnam National University, Gwangju, Republic of Korea; 2 Department of Biological Sciences, Chonnam National University, Gwangju, Republic of Korea; 3 Research Institute of Agriculture and Life Sciences, Seoul, Republic of Korea; 4 Suncheon Bay Conservation Division, Suncheon, Jeollanamdo, Republic of Korea; 5 National Natural Heritage Center, Daejeon, Republic of Korea; 6 Institute of Sustainable Ecological Environment, Chonnam National University, Gwangju, Republic of Korea; National Museums of Kenya Ornithology Section, KENYA

## Abstract

Understanding the spatial distribution of wintering birds in areas with interspecific competition is essential for the development of effective conservation and management strategies. This study investigated habitat use and resource partitioning in hooded cranes (*Grus monacha*) and geese (*Anser albifrons* and *Anser fabalis*) in Suncheon Bay, UNESCO World Natural Heritage Site. We specifically assessed the impact of habitat management strategies, particularly supplemental feeding, on the species distribution patterns and competition dynamics of hooded cranes and geese. Field surveys conducted from November 2022 to March 2023 revealed that hooded cranes consistently preferred site C-3, a protected area where rice grain is provided through conservation-focused management. In contrast, geese exhibited more adaptable habitat use, shifting their distribution in response to seasonal variations in food availability and the location of the hooded crane population. Utilization rates, electivity indices, and spatial niche analyses indicated that although both species initially overlapped in high-resource areas, geese expanded their spatial niche later in the season, leading to increased spatial separation. The gradual decline in niche overlap suggests resource partitioning as a strategy to reduce interspecific competition. These findings highlight the importance of managing avian conservation programs in a way that takes into account the need to maintaining availability and suitability of habitats for wintering species so as to promote interspecific coexistence amongst migratory bird populations.

## Introduction

Understanding how species partition resources and mitigate competition is a fundamental area of interest in ecology, influencing population dynamics, habitat use, and conservation strategies across taxa, ranging from avian species to large terrestrial mammals [1–3]. In temperate regions, migratory species experience seasonal fluctuations in resource availability, often resulting in interspecific competition that shapes spatial distribution and foraging strategies [4–6]. Although competition-driven niche partitioning has been widely documented in mammals such as ungulates [7–8] and carnivores [9], studies on long-distance migratory birds in human-altered landscapes are lacking.

In many migratory animals, spatiotemporal habitat shifts occur to minimize competition, particularly when resources become scarce [10]. For wintering birds that need to accumulate sufficient energy for long-distance migration, the availability of food resources during the wintering period significantly influences subsequent long-distance migration and breeding success [11–16]. According to the ideal free distribution (IFD) theory, the spatial distribution of wintering birds is influenced by the availability of prey resources within their wintering habitats [17–20]. However, it can also be influenced by other factors, such as the number of species and individuals arriving at the wintering grounds, as competition for limited food resources results in alterations in resource use among species and individuals [21–23]. Migratory birds that use agricultural land as their primary overwintering habitat often share habitats with other species, such as large birds belonging to the families Gruiformes and Anseriformes with similar ecological status and food resources (grain), leading to resource competition resources and overcrowding in these limited spaces [5,6,24,25]. In this scenario, competing individuals actively select foraging sites to minimize travel costs which is daily foraging-related movements within the wintering area and maximize the quality and quantity of food resources; therefore, overwintering individuals exhibit spatial habitat use patterns that improve foraging efficiency to facilitate their survival and reproductive success [1,6,20,26,27]. Hence, studies investigating species composition, spatial distribution, and movement within habitats are necessary for the protection and conservation of migratory birds, especially wintering species (including endangered species). This information is crucial for developing conservation measures, such as the establishment of protected areas and the provision of supplemental food resources [28–29].

Cranes, ducks, and geese primarily forage in agricultural [30–31] and feed on grain left over from human harvesting [20,32–35]. Cranes are generally omnivorous and rely heavily on grains and plant roots, whereas geese are primarily grazers feeding on grasses and winter pastures. These differences in trophic strategy influence their spatial overlap and interspecific interactions during wintering periods [6,36]. Grain management in agricultural lands is essential for species conservation, as the availability of grain directly affects the survival of wintering birds dependent on this resource [37–38]. Hence, artificial feeding is often used to ensure the availability of food resources in wintering habitats [38,39]. However, the implementation of such measures requires the cooperation of local residents. Therefore, various methods have been proposed to conserve ecosystem services, such as the payments for environmental services (PES) system, which provides economic compensation to residents to encourage their voluntary participation [40]. Similarly, in Korea, the Biodiversity Management Conservation Schemes (BMCS) program, led by the Ministry of Environment, provides food resources for wintering birds and conserves key wintering habitats through contractual agreements for ecosystem conservation. It also compensates residents for losses incurred due to government conservation efforts [41].

Earlier research on interspecific competition in birds largely focused on breeding colonies of resident species [42]. Migratory species have been comparatively underrepresented because their long-distance movements, transient presence across vast flyways, and large spatial ranges historically rendered both experimental manipulation and consistent observation logistically difficult [43–44]). This observational challenge often restricted studies of resource competition to smaller, more predictable populations. However, recent technological advancements have enabled several studies to be conducted. Recent investigations into large waterbird ecology have increasingly utilized advanced spatial modeling and tracking technologies to clarify multi-scale habitat selection and the impact of anthropogenic changes [16,27,45]). Studies focusing on the Grus and Anseriformes families confirm that habitat suitability is a complex product of environmental conditions (e.g., minimum temperature and distance to water) and land use characteristics, with many populations now relying heavily on agricultural fields for winter foraging [35,46,47]. This shift intensifies the potential for both intraspecific and interspecific competition in the limited agricultural and natural wetland patches that remain [6,29]). Furthermore, given the increasing reliance on cultivated lands, effective conservation strategies must incorporate detailed spatial data on resource availability and distribution, emphasizing the need for adaptive management protocols that account for dynamic resource partitioning throughout the wintering period.

Suncheon Bay, a coastal wetland designated as a UNESCO World Natural Heritage Site in the Republic of Korea, is a wintering habitat for a wide variety of bird species [48]. Various conservation policies are being implemented to preserve these populations [49]. The following wintering bird conservation policies are currently being implemented in Suncheon Bay and at the national level: (1) supplying food resources for wintering birds through the BMCS program and (2) establishing absolute protection zones where tourist access is restricted. Therefore, wintering bird populations mainly feed on rice grain in agricultural fields surrounding Suncheon Bay, resulting in interspecific competition for the use of wintering habitats due to a high concentration of bird populations within a limited area [24–25]). In Suncheon Bay, there are three representative species of winter migratory birds arrive during the winter: the hooded crane (*Grus monacha*), listed as a vulnerable species on the IUCN Red List, and goose species such as the white-fronted goose *Anser albifrons* and the bean goose *Anser fabalis.* The BMCS program plays a critical role in conserving wintering bird as well as shaping food competition; however, the extent to which interspecific competition influences spatial segregation remains unclear. Further investigation is required to determine whether hooded cranes actively displace geese or if geese adopt an opportunistic foraging strategy to avoid competition.

To address this gap in the literature, this study investigated the wintering dynamics of hooded cranes and geese in Suncheon Bay, with a particular focus on resource partitioning among these species and changes in foraging strategies over time. These species are common that they account for over 90% of the large waterbirds arriving at Suncheon Bay, and represent the longest cumulative individual bird-days that they frequently utilize nearby agriculture area [49]. We proposed three hypotheses regarding the spatial distribution of cranes and geese, each supported by recent ecological theory on habitat selection and interspecific coexistence (e.g., [50–51]). Predictions were added for each hypothesis to enhance testability, as follows:

Hypothesis 1

Habitat preference hypothesis Hooded cranes will preferentially use protected or low-disturbance areas and rely on anthropogenic grain resources.

### Prediction 1

Crane occurrence probability will be significantly higher in protected feeding sites than in open farmlands.

Hypothesis 2

Resource-use differentiation hypothesis Geese will mainly exploit open agricultural fields with natural vegetation, whereas cranes will concentrate at feeding grounds.

### Prediction 2

Spatial overlap indices between cranes and geese will decline with increasing distance from artificial feeding sites.

Hypothesis 3

Seasonal segregation and competition hypothesis As winter progresses and food resources diminish, both inter- and intraspecific competition among cranes intensifies at feeding sites. In response, geese—owing to their greater habitat flexibility and grazing ability—shift to alternative open fields, thereby reducing spatial overlap with cranes.

### Prediction 3

Niche overlap will be lower in late winter (February) than in early winter (December–January), reflecting increased intraspecific crowding among cranes and displacement of geese toward peripheral habitats.

By analyzing the seasonal habitat distribution and interactions between these species, this study provides insights into the ecological mechanisms driving wintering bird populations and highlights the importance of the implementation of the BMCS program in agricultural fields within the protected area for the effective management and conservation of wintering habitats.

## Materials and methods

### Study area

**Fig 1:**
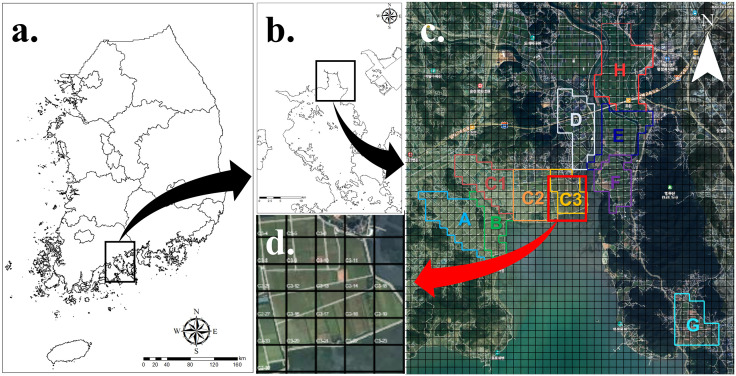
Research area and grid Information (a) Map of the Republic of Korea, (b) location of the Suncheon Bay, (c) The map of Suncheon Bay divided into sites A–H and further subdivided into 200 × 200 m grids, (d) Enlarged map of the site C-3. The base map data was sourced from the National Geographic Information Institute of Korea (NGII) and is open data for free use, with proper attribution given.

This study was conducted in agricultural areas surrounding the Suncheon Bay (34°8′N, 127°4′E; Fig 1), located in Suncheon-si, Jeollanam-do, the Republic of Korea. The survey was conducted from November 2022, when wintering birds began arriving in Suncheon Bay, to March 2023, when the birds migrated northward. This area is a representative wintering site for hooded cranes in Korea that hooded crane populations have increased substantially during the past decades: The number of hooded cranes has markedly increased since 2009, coinciding with the introduction of supplemental feeding by BMCS: approximately 350 individuals were recorded in 2010, and the wintering population grew to about 3,000 individuals by 2022 [49]. According to [49], hooded cranes using this area spend over 90% of their time in agricultural fields during the daytime; hence, we assumed that these fields are the primary foraging sites for wintering birds. For this study, the survey area was divided into 10 sites, with each site divided into a grid of 200 × 200 m to estimate the population of hooded cranes and geese at the subsite level (Fig 1). The number of grids was 50 for site A, 23 for site B, 37 for site C-1, 39 for site C-2, 23 for site C-3, 36 for site D, 43 for site E, 29 for site F, 31 for site G, and 55 for site H. At Sites A, B, C, D, and G, rice (*Oryza sativa*) is cultivated in paddy fields from spring to autumn, and no crops are grown from winter until the following spring. At Sites E, F, and H, rice is cultivated in paddy fields from spring to autumn, and bulky feed for livestock are grown from winter until the following spring

Since 2009, Suncheon Bay has designated 62 ha areas managed through the BMCS program annually, restricting human access during winter and supplying food resources for hooded cranes [49]. Site C-3, which is managed under the BMCS program, is referred to as the Hope Agricultural Complex and is primarily used for rice cultivation, producing an average of 250 t of rice grain per year. Rice is a highly nutritious crop, serving as a major food resource for wintering birds in agricultural areas [52–53]). Of the 250 t of grain cultivated in the C-3 site, approximately 158 t was directly distributed by leaving them onto the cropland following the harvest in November. The remaining 92 tons were stored in warehouses and supplied at a rate of 8 tons per week starting from the first week of January. The spatial extent of the feeding area in the site C-3 was approximately 400 × 200 m (about 4 grid squares), with different feeding locations designated each week on a rotating basis within the 62 ha protected area.

### Field survey

Weekly surveys were conducted from the first week of November 2022 to the fourth week of March 2023 to investigate the spatial distribution of wintering birds. Surveys were performed twice per survey day, once in the morning and once in the afternoon. Morning surveys were conducted two hours after sunrise, whereas afternoon surveys were conducted 2.5 hours before sunset, with each survey lasting 120 min. Two survey teams, each consisting of two trained observers, conducted simultaneous counts to maximize coverage and minimize duplicate observations. The teams communicated via radio to prevent double-counting individuals moving between survey areas. The study area was divided into predefined wintering sites for hooded cranes and geese, ensuring systematic coverage of their preferred habitats. Bird distribution was assessed using the line transect method, with observers scanning designated survey grids using binoculars (Endeavor ED II 8 × 42, Vanguard) and a field scope (ATX 25−60 × 85, Swarovski). A vehicle-based approach was employed to improve survey efficiency and minimize disturbance, allowing observers to move systematically between transects. Key observation points, such as the C-3 transect at Yongsan Observatory (34°8′N, 127°5′E), provided elevated vantage points for counting birds across large areas. To ensure count accuracy, sight ability factors were considered based on environmental conditions such as weather, visibility, and light conditions. Each survey site was delimited by natural topographical features to maintain consistency across repeated counts.

### Data analysis

Population data on the distribution of hooded cranes and geese were collected from 366 grids across 10 survey areas. The number of hooded cranes counted each week was calculated as the average of the morning and afternoon counts. The spatial distribution of hooded cranes and geese was plotted on a 200 × 200 m grid using ArcMap 10.1 (Esri, USA) and expressed as the average number of individuals per month during the wintering season.

To determine the monthly distribution of hooded cranes and geese by site, the utilization rate proposed by [6] was used. The utilization rate (Ui) represents the preference pattern based on the number of individuals present at each site and was calculated as follows:

Ui = Ni/N,

where Ui is the utilization rate of the *ith* site for hooded cranes and geese, Ni is the number of wintering birds at the *ith* site, and N is the total number of wintering birds across all sites. The utilization rate of hooded cranes is presented as the mean ± SD (n = number of surveys) for all sites with monthly variations.

Spatial niches were calculated based on the distribution data obtained from the surveys. The width of the spatial niches was calculated using the Shannon–Wiener diversity [23,54]) as follows:

*B*_i_ = −Σ*P*_i_ ln*P*_i_,

where Bi is the width of the niche and Pi is the percentage value of the observed abundance of the species in the ith habitat type relative to the total observed abundance of the species.

The degree of spatial niche overlap between overwintering species in Suncheon Bay was calculated using the following Pianka’s (1974) equation ([6,23]).

*O*_ij_ = Σ*P*_ik_*P*_jk_/(Σ*P*_ik_^2^ Σ*P*_jk_^2^)^½^

The percentage of the two species – hooded crane (i) and geese (j) – observed in the kth habitat type, where an Oij value of 0 indicates no niche overlap and an Oij value of 1 indicates complete niche overlap.

The relative site use indicator, based on Ivlev’s electivity index, was used to assess site preference for each species [55–57]. Site use and preference were determined by calculating the percentage of grids occupied by crane and goose populations relative to the total number of grids. Therefore, the electivity index represents the distribution of the entire population, as it indicates preference even when a single individual is present in the grid. The electivity index was computed using the formula S = (a − b)/(a + b), where S represents the electivity index, a represents the percentage of grids used by wintering bird flocks in the sector, and b denotes the percentage of the specific habitat area relative to the total number of available [57]. For each site, electivity values ranged from −1.0 (never used) to +1.0 (exclusively used), with positive and negative electivity values denoting site preference and avoidance, respectively [55,57]. Monthly variations in site preference for each species were also assessed throughout the survey period.

Statistical analyses were conducted using SPSS (Version 21, IBM Corp.) The Mann-Whitney U test was used to evaluate the effectiveness of BMCS-implemented anthropogenic feeding for hooded cranes and geese. Pearson’s correlation analysis was used to examine the relationship between spatial niche overlap and week progression. To evaluate the effects of interspecific competition and habitat factors on the distribution patterns of wintering hooded cranes and geese, we used linear mixed models (LMMs). LMMs were used to analyze variations in habitat preference across sites, considering species (hooded cranes vs. geese), month, and presence of anthropogenic food sources (BMCS implementation area) as fixed effects. Site ID was included as a random effect to account for repeated measures across different locations. The significance of the fixed effects was tested using a Type III ANOVA, with the significance level set at 0.05. LMM analyses were conducted using the R statistical environment (version 4.3.1; R Foundation for Statistical Computing, Vienna, Austria), utilizing the *lme4* package for model fitting and the *lmerTest* package for the calculation of coefficients and p-values [58].

### Ethical note

This study followed the guidelines established by the Guide for the Care and Use of Laboratory Animals (Institute of Laboratory Animal Resources 1986). All procedures complied with Korean laws and were conducted in accordance with permits issued by the Suncheon Bay Conservation Division, Suncheon City Hall, in the Republic of Korea (Permission number: 2022-4041-01) and the approval of the Institutional Animal Care and Use Committee of Chonnam National University (Permission number: 21.6.24).

## Results

### Changes in the populations of hooded cranes and geese during the wintering season

**Fig 2:**
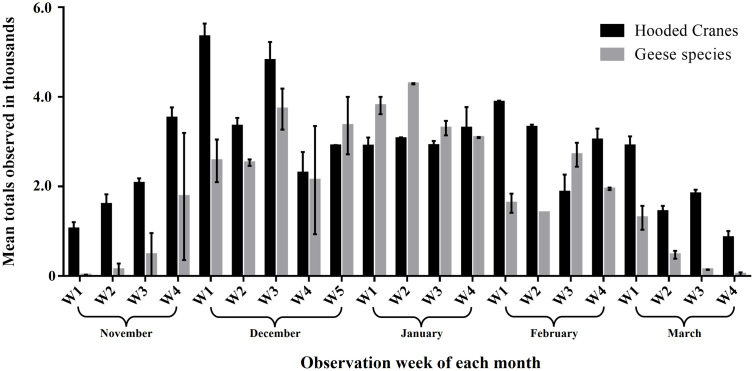
Changes in the wintering populations of hooded cranes and geese in Suncheon Bay (November 2022–March 2023). The hooded crane population peaked in the first week of December, with approximately 3,000 individuals observed overwintering. The geese arrived later than the hooded cranes, peaking in the second week of January before showing a declining trend.

Among the birds overwintering in Suncheon Bay, cranes began arriving in the first week of November. A total of 2,649 ± 242 individuals (mean ± SE) overwintered in Suncheon Bay area, remaining until the fourth week of March, when they migrated northward. The highest number of hooded cranes in Suncheon Bay was observed during the first week of December (average: 5,112 individuals) when individuals southbound to Izumi joined the population. The population remained stable at approximately 3,000 individuals until the first week of March, after which it increased with the arrival of northbound individuals from Izumi around late February. By the fourth week of March, all hooded cranes had completed their northward migration. Goose species began arriving in considerable numbers in the first week of December, approximately one month later than the hooded cranes. The highest number of individuals was observed in the second week of January (average: 4,294 individuals). The goose species commenced their northward migration in late February, earlier than the hooded cranes, completing their northward migration by the end of March (Fig 2).

### Distribution patterns of wintering birds in Suncheon Bay

**Fig 3:**
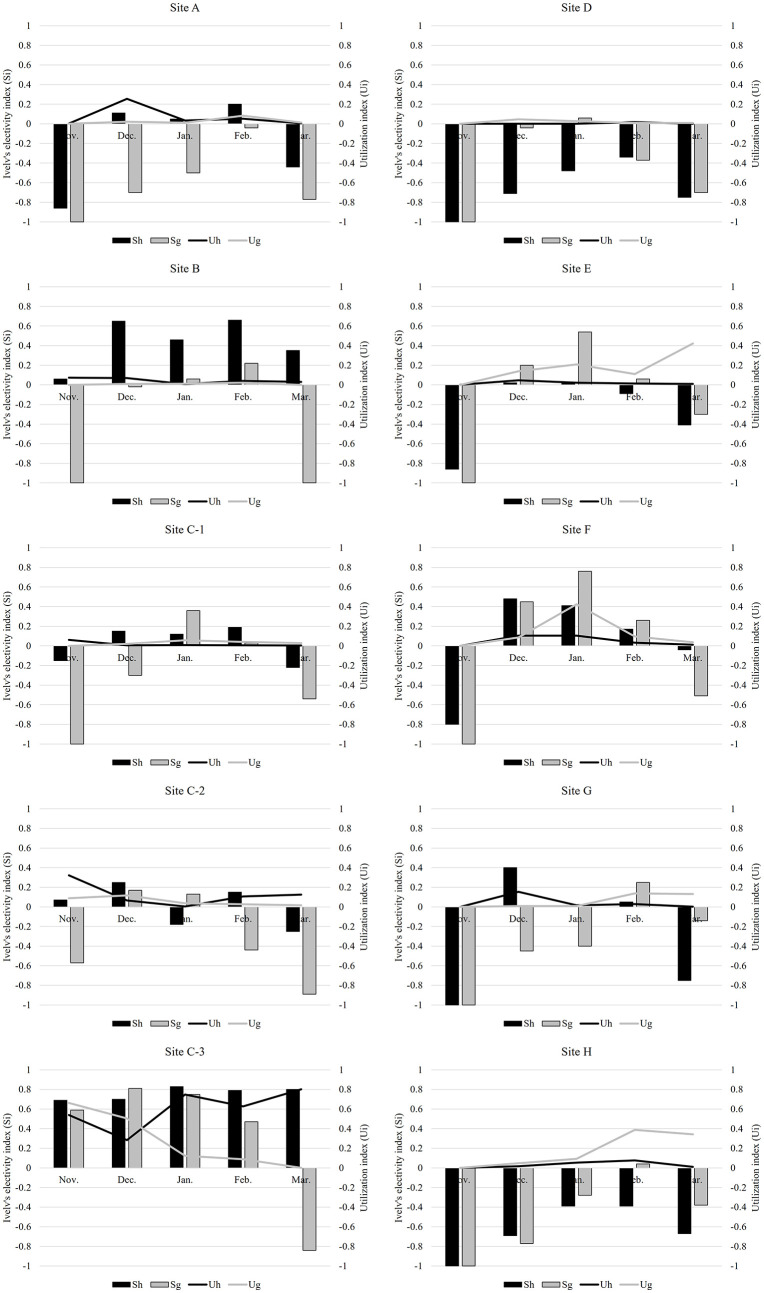
Monthly variations in site preferences based on Ivlev’s electivity index (hooded cranes: Sh; geese: Sg) and utilization rate (hooded cranes: Uh, geese: Ug) in Suncheon Bay, Republic of Korea. Bar graphs represent Ivlev’s electivity index, with positive values indicating site preference and negative values indicating site avoidance. Line graphs show utilization rates reflecting habitat utilization by hooded cranes and geese species.

During the wintering season in Suncheon Bay, hooded cranes primarily used site C-3, where the BMCS program was implemented. The average utilization rate of hooded cranes was highest at site C-3 (0.60 ± 0.18, *n* = 42), followed by site C-2 (0.12 ± 0.11, *n* = 42) and site A (0.07 ± 0.09, *n* = 42). Site C-3 recorded the highest utilization rate each month, followed by site C-2 (0.32 ± 0.27, *n* = 8) in November, site A (0.25 ± 0.20, n = 10) in December, site F (0.10 ± 0.18, *n* = 8) in January, site C-2 (0.11 ± 0.12, *n* = 8) in February, and site C-2 (0.13 ± 0.13, n = 8) in March. Furthermore, for geese, site C-3, where the BMCS program was implemented, was the most used site (Table 1). The average utilization rate was highest at site C-3 (0.28 ± 0.26, n = 42), followed by site E (0.18 ± 0.14, *n* = 42) and site H (0.17 ± 0.16, n = 42). At the beginning of the wintering season, the utilization rate was highest at site C-3 in November and December (November: 0.66 ± 0.47, *n* = 8; December: 0.50 ± 0.21, *n* = 10). After the mid-wintering season, the area with the highest utilization rate varied monthly (site F in January: 0.42 ± 0.08, *n* = 10; site H in February: 0.39 ± 0.21, *n* = 8; and site E in March: 0.42 ± 0.32, n = 8). The presence of anthropogenic food sources significantly affected hooded crane site preference (Mann-Whitney U test; *Z* = 3.92, *p* < 0.01). In contrast, geese exhibited greater flexibility in habitat selection as their distribution was not affected by anthropogenic food source availability (Mann-Whitney U test; *Z* = 0.98, *p* = 0.324), leading to a shift in their distribution later in the season (Fig 3).

**Table 1:** Impact of BMCS and seasonal timing on habitat utilization. The Linear Mixed Model (LMM) was employed to assess the effects of supplemental feeding (BMCS), species, and time on habitat utilization intensity ($Ivelv\ index$), controlling for repeated measures at the site level.

Predictor	Estimate (β)	SE	t-value	p-value
(Intercept)	−0.86	0.147	−5.856	< 0.001*
BMCS	0.822	0.18	4.562	< 0.001*
Species	−0.14	0.176	−0.793	0.429
Time	0.42	0.187	2.243	0.026*
Species × BMCS	−0.178	0.224	−0.796	0.427
Time × BMCS	−0.495	0.257	−1.925	0.056
Species × Time × BMCS	−1.075	0.364	−2.956	0.004*
Random effect (Site)	σ2 = 0.0216	—	—	—

**Fig 4:**
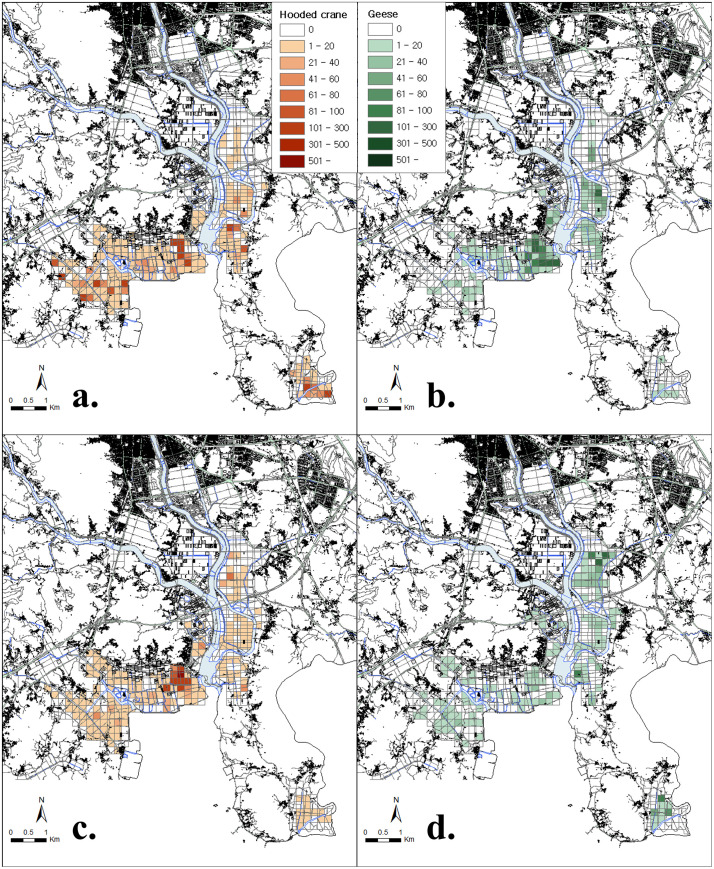
Comparison of the distribution range of the monthly average numbers of hooded cranes and geese between December 2022 and February 2023 in Suncheon Bay; (a) hooded cranes in December, (b) geese in December, (c) hooded cranes in February, and (d) geese in February. The base map data was sourced from the National Geographic Information Institute of Korea (NGII) and is open data for free use, with proper attribution given.

**Fig 5:**
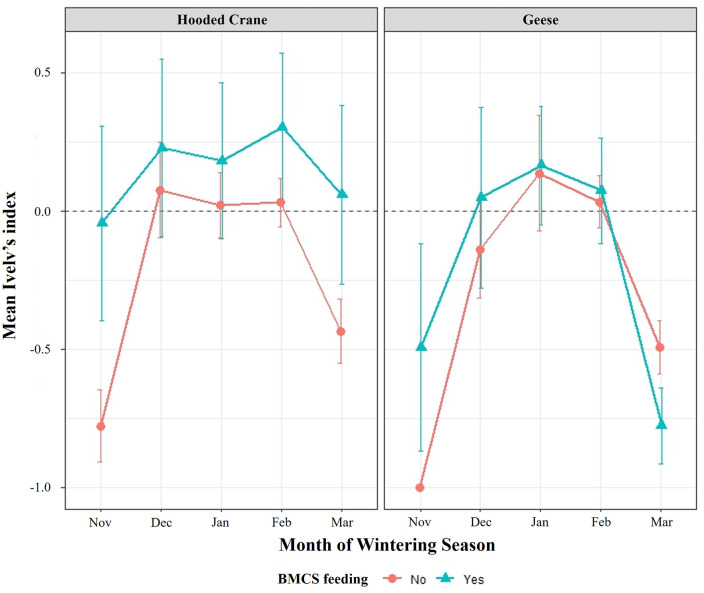
Monthly variation in habitat utilization index (Ivelv’s index) for Hooded Cranes and Geese across BMCS and non-BMCS sites. Hooded Cranes maintain a consistently high Ivelv’s index at BMCS sites throughout the winter. In contrast, Geese show high utilization at BMCS sites early in the season, but this utilization drops sharply in March.

Both hooded cranes and geese exhibited a preference for site C-3 (positive selection), where the BMCS program was implemented; however, the electivity indices showed monthly variations (Fig 3). Hooded cranes showed a consistent positive preference for sites C-3 and B during the wintering period and a continuous negative preference for sites D and H. During the mid-winter period, the preference for sites A, C-1, and F increased. Habitat preferences changed rapidly in November and March, i.e., the early and late winter months, when significant population shifts occurred. In contrast, geese showed dynamic site preferences throughout the wintering period, consistently shifting between sites C-2, C-1, E, F, and G. The habitat use intensity was significantly influenced by the presence of the feeding program (BMCS: F = 22.083, P < 0.001) and the month of observation (Time: F = 11.08, P < 0.001). The main effect of species was not statistically significant (F = 1.348, P = 0.247). We found the highly significant three-way interaction between species, time, and BMCS presence (F = 3.864, P = 0.005). This result indicates that the effect of the supplemental feeding program on habitat use intensity varied significantly depending on the species and the stage of the wintering season(Table 1, Figs 4, 5).

### Spatial niche width and overlap between hooded cranes and geese

**Fig 6:**
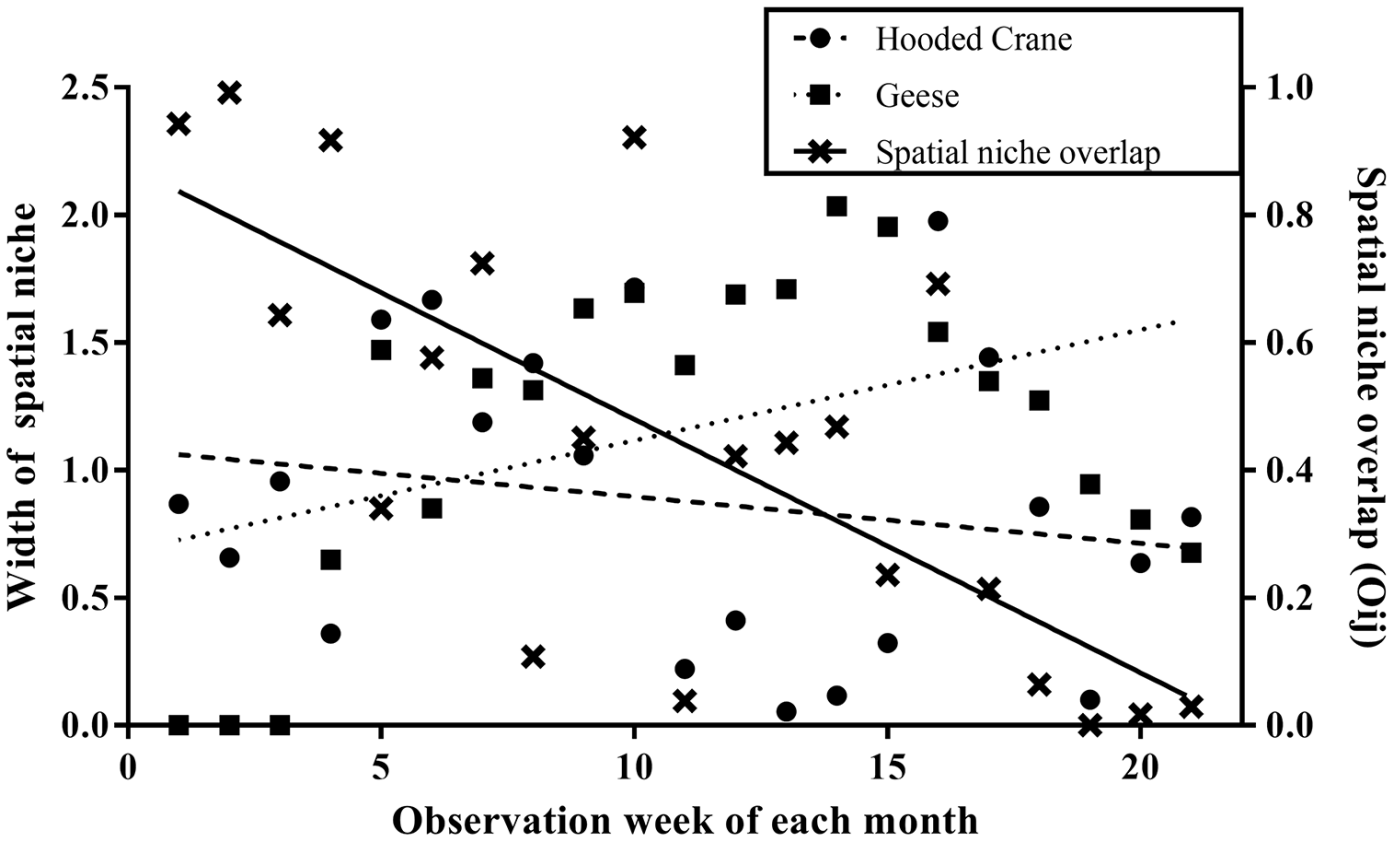
Spatio-temporal dynamics of habitat utilization by hooded cranes and geese throughout the wintering period. High utilization and overlap in Early Winter reflect resource abundance (including BMCS-provisioned sites). The subsequent divergence and decreased utilization in Mid- to Late Winter indicate increasing interspecific competition and resource partitioning, driving Geese to shift distribution away from the primary feeding area.

In November and December, hooded cranes exhibited a broader spatial niche compared to geese. However, from January through March, the width of the spatial niche of geese was greater than that of hooded cranes (Table 2). The width of the spatial niche width of geese significantly increased over time (*r*_*p*_ = 0.433, *n* = 21, *p* < 0.05), whereas that of hooded cranes gradually decreased; however, this trend was not statistically significant (*rp* = −0.192, *n* = 21, *p* > 0.05) (Fig 6). The degree of spatial niche overlap between hooded cranes and geese was highest in November, with a significant decrease observed by week as winter progressed (*r_p_* = −0.730, *n* = 21, *p* < 0.001) (Table 2, Fig 6).

**Table 2:** Comparison of spatial niche width and spatial niche overlap (Oij) between hooded cranes and geese during the wintering season.

	Width of spatial niche				
	November	December	January	February	March
Hooded cranes	0.67	1.84	0.95	1.15	0.78
Geese	0.57	1.62	1.68	1.81	1.32
Spatial niche overlap (Oij)	0.99	0.74	0.35	0.26	0.03

## Discussion

Wintering birds are exposed to intraspecific and interspecific competition due to the limited availability of food resources and the high density of individuals in a limited space during the wintering period [22,23,59]. Moreover, variations in spatiotemporal habitat distribution observed among species and individuals maximize food utilization during this period [60]. In colonial waterbirds, food resource availability significantly influences flock dynamics, driving changes in the niche dynamics of wintering populations [61–62]. Our findings support the hypothesis that interspecific competition influences habitat selection, leading to spatial separation between hooded cranes and geese as food resources decline. In this study, both species initially exhibited a preference for high-resource areas, particularly sites where the BMCS program was implemented. However, as winter progressed, geese exhibited a more adaptable habitat use pattern, shifting to non-protected areas, whereas hooded cranes remained strongly associated with anthropogenic food sources.

The habitat usage of wintering birds is closely linked to food availability [15,63]. When food resources are less abundant, wintering birds migrate and disperse, adopting flexible foraging strategies based on the availability of food resources within a limited habitat [23,64,65]. In this study, the utilization rate of hooded cranes was consistently high at site C-3 throughout the wintering season. In contrast, the utilization rate of geese varied and tended to extend outward from site C-3. Habitat preference analysis based on Ivlev’s electivity index revealed that hooded cranes consistently preferred site C-3 from the beginning to the end of the wintering period, occasionally moving to other areas, such as sites A, B, and F. Conversely, geese exhibited a relatively dynamic habitat preference during the wintering period. In terms of the distribution, differences were observed between the utilization index and the electivity index for the hooded crane population, whereas geese exhibited a consistent pattern. As food resources decrease during overwintering, bird populations are concentrated in a limited space to forage for available food resources, resulting in increased competition [6,66] This supports our first and second hypotheses, which posits that hooded cranes prefer protected areas with anthropogenic food resources, whereas geese demonstrate greater habitat flexibility. When artificial feeding occurs, migration patterns differ from those in natural conditions, and migration patterns vary due to differences in preferred food between cranes and geese. However, both cranes and geese show a tendency to prioritize rice as their most nutritious food source that the initial distribution of both species overlapped in BMCS-protected fields with abundant food resources. However, as the wintering season progressed, hooded cranes remained concentrated in priority protected sites (e.g., C-3), whereas the goose population shifted to peripheral areas (A, B, and F). This suggests that cranes may secure food resources more effectively than geese, potentially leading to spatial separation, while geese, capable of consuming a wider variety of foods, may migrate to other areas to avoid competition. Thus, as the wintering period progressed, geese may have adopted a strategy of avoiding areas with high hooded crane densities to reduce interspecific competition by using alternative sites.

Increases in population size in habitats with limited food resources lead to intensified intraspecific competition for food, which affects population distribution [67–68]. Competition inevitably arises due to the need to utilize limited food resources within a limited space, leading to spatial partitioning as a strategy to minimize overlap and maximize the utilization of food resources [4,5,23] In this study, changes in habitat use were further reflected in the width of the spatial niche. During the early winter months (November–December), hooded cranes exhibited a broader spatial niche compared to geese. However, from January onward, geese expanded their habitat use, resulting in a wider niche breadth than that of hooded cranes. As mentioned earlier, hooded cranes primarily rely on rice as their food source during the wintering period, while geese can consume a variety of food sources including rice (such as plant roots). As rice stocks deplete across all regions, hooded cranes become dependent on artificial rice feeding, concentrating their population distribution within protected areas. Geese, however, disperse to other areas to access alternative food sources. Particularly in February and March, bulky feed sprouts emerge, allowing geese to forage at sites E, F, and H. The increasing spatial niche width in geese and the non-significant decrease in hooded cranes indicate that geese adapted more dynamically to food resource changes as winter progressed. This finding supports our third hypothesis, which suggests that the distribution of both species shifts over the wintering period, with an initial overlap in high-resource areas followed by increasing spatial separation.

The spatial niche overlap between hooded cranes and geese was highest in November but significantly decreased over time. This trend aligns with the principle that interspecific competition drives resource partitioning. Initially, both species occupied similar habitats; however, as winter progressed, competition likely intensified due to declining natural food availability and supplement of artificial feeding, leading to spatial separation. The significant shift of goose populations towards non-protected areas suggests that they may be more adaptable in their foraging strategies, whereas hooded cranes rely more heavily on stable food resources in protected areas. [64] reported that wintering birds moved between habitats depending on food resource availability to maximize resource utilization. In Suncheon Bay, crops in agricultural fields were primarily consumed during the peak arrival period in December. Hooded cranes in this region dispersed to areas outside the priority protected area to feed. At this time, geese [69], which consume less food per individual, benefited from the absence of hooded cranes in the priority protected area and moved to feed on the remaining food at site C-3 [49]. After January, when anthropogenic feeding was introduced, the hooded crane population preferentially occupied the priority protected areas, whereas geese moved to areas with lower hooded crane densities. Therefore, the distribution of geese during the wintering period is considered an opportunistic foraging strategy [6,70] observed that hooded cranes and geese arriving at Shengjin Lake in China exhibited a gradual increase in niche overlap due to the limited availability of food resources during the late wintering period. The decline in natural food sources resulted in increased resource partitioning. However, after the implementation of artificial feeding, the spatial niche overlap between hooded crane and goose populations decreased toward the end of the wintering season. Similarly, hooded cranes and geese arriving at Suncheon Bay exhibited resource utilization partitioning, a behavior characteristic of coexisting species, by segregating their habitat use areas through spatial separation. This trend may be attributed to interspecific competition between the two species. Spatial constraints in the feeding area since January, when anthropogenic feeding was introduced, may have resulted in some goose species being outcompeted for food, leading to their dispersal to surrounding areas. Therefore, differences in habitat use between species are explained by ‘competitive avoidance’. Hooded cranes maintain a choice to remain at the best resource (BMCS site) throughout the season based on its high dominance there. However, geese initially utilize BMCS sites but later in winter, when competitive pressure increases, they reduce their use of BMCS sites and disperse to other farmlands, demonstrating selective avoidance. Therefore, this results demonstrates that differential habitat use arises through interspecific competitive dynamics, influenced by the presence or absence of BMCS and the passage of time.

Anthropogenic feeding programs can have complex ecological effects that extend beyond immediate population benefits. In the short term, supplemental feeding enhances overwinter survival, reduces starvation risk, and can stabilize local population to wintering birds [71–72]. However, continuous reliance on anthropogenic food sources may also alter natural foraging behavior, reduce habitat use diversity, and increase the likelihood of intraspecific competition and aggressive interactions at feeding sites [73]. In addition, dense aggregations of birds around concentrated feeding areas can facilitate disease transmission, and increase parasite loads, such as avian influenza [74–75]. Over longer timescales, such programs may induce phenological shifts—such as delayed migration or changes in site fidelity—that could compromise species’ resilience to environmental variability [73,76]. For effective conservation management, it is therefore crucial to balance the short-term benefits of artificial feeding with potential ecological costs by periodically evaluating the timing, quantity, and spatial allocation of food provisioning.

The environmental carrying capacity of wintering sites is influenced by multiple factors, such as disturbance, food resource availability, interspecific competition, and the environmental conditions of breeding sites [77–78]. For large birds that breed in agricultural areas, human access and disturbance pose significant threats to their habitat. Currently, policies aimed at mitigating these threats vary globally, aiming to provide adequate and safe foraging and resting areas [79–80]. The BMCS policy, a contractual agreement between the government and local residents, aims to promote the conservation of ecologically significant areas. This policy encourages the voluntary participation of local residents engaged in direct farming to promote coexistence between humans and nature [41]. In Suncheon City, the designation of the Suncheon Bay Wetland Reserve prohibits human access to wintering habitats of birds and provides continuous food resources for wintering birds in accordance with BMCS. Therefore, the number of overwintering birds fluctuates in response to the environmental conditions and food resource availability at site C-3 of Suncheon Bay. This site provides stable food resources for cranes but also creates a competitive environment for geese [81], resulting in high hooded crane densities in protected fields and potentially increasing competition for geese. Future conservation efforts should consider optimizing food distribution to reduce interspecific competition while maintaining stable wintering populations.

In conclusion, this study provides valuable insights into the dynamics of wintering bird populations, focusing on hooded cranes and geese in Suncheon Bay, South Korea. This study, which focuses on the impact of the BMCS program, elucidates the impact of anthropogenic feeding on the distribution patterns and interspecific competition dynamics of these avian species. Hooded cranes occupied high-quality foraging sites, displacing geese to less preferred areas (H1). However, geese exhibited opportunistic foraging behavior, adjusting their habitat use based on resource availability and crane distribution (H2). These findings align with the broader ecological principle that species dynamically partition resources to minimize competition (H3). This study suggests that sustained habitat protection, combined with the provision of food resources, remains crucial for the effective management of wintering habitats and the conservation of endangered species, particularly the hooded crane. The observed resource partitioning between hooded cranes and geese as winter progresses highlights the need for adopting a holistic and adaptive approach to conservation strategies. Furthermore, the study findings highlight the importance of considering anthropogenic influences when formulating conservation policies, particularly in areas where human activities and wildlife conservation overlap. Continuous ecological research and a dynamic conservation framework are essential to address evolving challenges and ensure the long-term viability of wintering bird populations in Suncheon Bay and similar ecosystems worldwide.

## References

[1] Schoener TW. Resource partitioning in ecological communities: Research on how similar species divide resources helps reveal the natural regulation of species diversity. Science. 1974;185(4145):27–39. 17779277 10.1126/science.185.4145.27

[2] Tilman D. Resource competition and community structure. Princeton University Press; 1982. 7162524

[3] Santangeli A, Mammola S, Lehikoinen A, Rajasärkkä A, Lindén A, Saastamoinen M. The effects of protected areas on the ecological niches of birds and mammals. Sci Rep. 2022;12(1):11601. doi: 10.1038/s41598-022-15949-2 35804004 PMC9270413

[4] Martin TE, Karr JR. Behavioral plasticity of foraging maneuvers of migratory warblers: multiple selection periods for niches. Studies in Avian Biology. 1990;13(1):48.

[5] Moore FrankR, Yong W. Evidence of food-based competition among passerine migrants during stopover. Behav Ecol Sociobiol. 1991;28(2). doi: 10.1007/bf00180984

[6] Zhao F, Zhou L, Xu W. Habitat utilization and resource partitioning of wintering Hooded Cranes and three goose species at Shengjin Lake. CHINESE BIRDS. 2013;4(4):281–90. doi: 10.5122/cbirds.2013.0032

[7] Dutoit JT. Feeding‐height stratification among African browsing ruminants. African Journal of Ecology. 1990;28(1):55–61. doi: 10.1111/j.1365-2028.1990.tb01136.x

[8] Arsenault R, Owen‐Smith N. Facilitation versus competition in grazing herbivore assemblages. Oikos. 2002;97(3):313–8. doi: 10.1034/j.1600-0706.2002.970301.x

[9] Hayward MW, Kerley GIH. Prey preferences and dietary overlap amongst Africa’s large predators. South African Journal of Wildlife Research. 2008;38(2):93–108. doi: 10.3957/0379-4369-38.2.93

[10] Macarthur R, Levins R. The Limiting Similarity, Convergence, and Divergence of Coexisting Species. The American Naturalist. 1967;101(921):377–85. doi: 10.1086/282505

[11] Tacha TC, Vohs PA, Iverson GC. Time and Energy Budgets of Sandhill Cranes from Mid-Continental North America. The Journal of Wildlife Management. 1987;51(2):440. doi: 10.2307/3801032

[12] Fredrickson LH, Reid FA. Waterfowl management handbook. 1988.

[13] Heaney V, Monaghan P. Optimal allocation of effort between reproductive phases: the trade-off between incubation costs and subsequent brood rearing capacity. Proceedings of the Royal Society of London Series B: Biological Sciences. 1996;263(1377):1719–24.

[14] Guy Morrison RI, Davidson NC, Wilson JR. Survival of the fattest: body stores on migration and survival in red knots C alidris canutus islandica. Journal of Avian Biology. 2007;38(4):479–87. doi: 10.1111/j.0908-8857.2007.03934.x

[15] Pearse AT, Selbo SM. Model of whooping crane energetics as foundation for development of a method to assess potential take during migration. US Geological Survey; 2012.

[16] Askren RJ, Massey ER, James JD, Osborne DC. Migration chronology and multi-scale habitat selection of wintering midcontinent greater white-fronted geese. Global Ecology and Conservation. 2022;39:e02290. doi: 10.1016/j.gecco.2022.e02290

[17] Emlen JM. The Role of Time and Energy in Food Preference. The American Naturalist. 1966;100(916):611–7. doi: 10.1086/282455

[18] MacArthur RH, Pianka ER. On Optimal Use of a Patchy Environment. The American Naturalist. 1966;100(916):603–9. doi: 10.1086/282454

[19] Fretwell SD, Lucas HL Jr. On territorial behavior and other factors influencing habitat distribution in birds. Acta Biotheor. 1969;19(1):16–36. doi: 10.1007/bf01601953

[20] Zheng M, Zhou L, Zhao N, Xu W. Effects of variation in food resources on foraging habitat use by wintering Hooded Cranes (Grus monacha). Avian Res. 2015;6(1). doi: 10.1186/s40657-015-0020-3

[21] Goss-Custard JD. Competition for Food and Interference among Waders. Ardea. 2002;38–90:31–52. doi: 10.5253/arde.v68.p31

[22] Martin TE. Food as a limit on breeding birds: a life-history perspective. Annu Rev Ecol Syst. 1987;18(1):453–87. doi: 10.1146/annurev.es.18.110187.002321

[23] Kober K, Bairlein F. Habitat Choice and Niche Characteristics Under Poor Food Conditions. A Study on Migratory Nearctic Shorebirds in the Intertidal Flats of Brazil. Ardea. 2009;97(1):31–42. doi: 10.5253/078.097.0105

[24] Zhou B, Zhou L, Chen J, Cheng Y, Xu W. Diurnal time-activity budgets of wintering Hooded Cranes (Grus monacha) in Shengjin Lake, China. Waterbirds. 2010;:110–5.

[25] Fox AD, Lei C, Barter M, Rees EC, Hearn RD, Hao CP, et al. The functional use of East Dongting Lake, China, by wintering geese. Wildfowl. 2013;58(58):3–19.

[26] Dingle H, Drake VA. What is migration? Bioscience. 2007;57(2):113–21.

[27] Overton CT, Casazza ML. Movement behavior, habitat selection, and functional responses to habitat availability among four species of wintering waterfowl in California. Front Ecol Evol. 2023;11. doi: 10.3389/fevo.2023.1232704

[28] Cañadas A, Sagarminaga R, De Stephanis R, Urquiola E, Hammond PS. Habitat preference modelling as a conservation tool: proposals for marine protected areas for cetaceans in southern Spanish waters. Aquatic Conservation. 2005;15(5):495–521. doi: 10.1002/aqc.689

[29] Corriveau A, Klaassen M, Garnett ST, Kaestli M, Christian K, Crewe TL, et al. Seasonal space use and habitat selection in magpie geese: implications for reducing human‐wildlife conflicts. J Wildl Manag. 2022;86(7). doi: 10.1002/jwmg.22289

[30] Kim M-R, Nam H-K, Kim M-H, Cho K-J, Kang K-K, Na Y-E. Status of Birds Using a Rice Paddy in South Korea. Korean Journal of Environmental Agriculture. 2013;32(2):155–65. doi: 10.5338/kjea.2013.32.2.155

[31] Fox AD, Elmberg J, Tombre IM, Hessel R. Agriculture and herbivorous waterfowl: a review of the scientific basis for improved management. Biol Rev Camb Philos Soc. 2017;92(2):854–77. doi: 10.1111/brv.12258 26946181

[32] Krapu GL, Facey DE, Fritzell EK, Johnson DH. Habitat Use by Migrant Sandhill Cranes in Nebraska. The Journal of Wildlife Management. 1984;48(2):407. doi: 10.2307/3801172

[33] Reinecke KJ, Krapu GL. Feeding Ecology of Sandhill Cranes during Spring Migration in Nebraska. The Journal of Wildlife Management. 1986;50(1):71. doi: 10.2307/3801490

[34] Sherfy MH, Anteau MJ, Bishop AA. Agricultural practices and residual corn during spring crane and waterfowl migration in Nebraska. J Wildl Manag. 2011;75(5):995–1003. doi: 10.1002/jwmg.157

[35] Thornton MJ, Mitchell C, Griffin LR, Briers RA, Minshull B, Maciver A, et al. Multi-scale habitat selection and spatial analysis reveals a mismatch between the wintering distribution of a threatened population of Taiga Bean Geese Anser fabalis and its protected area. Bird Study. 2021;68(2):157–73. doi: 10.1080/00063657.2021.1966740

[36] Monti F, Ferretti F, Fattorini N. Intrinsic and extrinsic factors modulating vigilance and foraging in two gregarious foragers. Behavioral Ecology. 2024;35(1). doi: 10.1093/beheco/arad114

[37] Siriwardena GM, Baillie SR, Wilson JD. Variation in the survival rates of some British passerines with respect to their population trends on farmland. Bird Study. 1998;45(3):276–92. doi: 10.1080/00063659809461099

[38] Siriwardena GM, Calbrade NA, Vickery JA, Sutherland WJ. The effect of the spatial distribution of winter seed food resources on their use by farmland birds. Journal of Applied Ecology. 2006;43(4):628–39. doi: 10.1111/j.1365-2664.2006.01170.x

[39] Siriwardena GM, Stevens DK, Anderson GQA, Vickery JA, Calbrade NA, Dodd S. The effect of supplementary winter seed food on breeding populations of farmland birds: evidence from two large‐scale experiments. Journal of Applied Ecology. 2007;44(5):920–32. doi: 10.1111/j.1365-2664.2007.01339.x

[40] Wunder S, Engel S, Pagiola S. Taking stock: A comparative analysis of payments for environmental services programs in developed and developing countries. Ecological Economics. 2008;65(4):834–52. doi: 10.1016/j.ecolecon.2008.03.010

[41] Yoo J, Yeo S, Kong K. Economic evaluation on the biodiversity management contract scheme. Korean Journal of Agricultural Management and Policy. 2012;39(2).

[42] Dhondt AA. Interspecific competition in birds. Oxford University Press; 2012.

[43] Zöckler C. Migratory bird species as indicators for the state of the environment. Biodiversity. 2005;6(3):7–13. doi: 10.1080/14888386.2005.9712769

[44] Faaborg J, Holmes RT, Anders AD, Bildstein KL, Dugger KM, Gauthreaux SA Jr, et al. Conserving migratory land birds in the new world: do we know enough?. Ecol Appl. 2010;20(2):398–418. doi: 10.1890/09-0397.1 20405795

[45] Sun X, Zhou L, Zhang Z, Meng L. Spatio-Temporal Distribution Patterns and Determinant Factors of Wintering Hooded Cranes (Grus monacha) Population. Diversity. 2022;14(12):1091. doi: 10.3390/d14121091

[46] Jiang Z, Shao M, Wang J. Simulation of Spatial and Temporal Patterns of Suitable Wintering Habitat for Hooded Crane (Grus monacha) Under Climate and Land Use Change Scenarios. Animals (Basel). 2024;15(1):6. doi: 10.3390/ani15010006 39794949 PMC11718936

[47] Wang J, Shao M. Regional differences in wintering habitat selection strategies of Siberian Crane (Leucogeranus leucogeranus) and ecological network construction of key wintering areas. Avian Research. 2025;16(3):100273. doi: 10.1016/j.avrs.2025.100273

[48] World heritage list. Getbol, Korean tidal flats. UNESCO World Heritage Centre; 2021. https://whcunesco.org/en/decisions/7925

[49] Suncheon. Habitat management of hooded cranes in Suncheon Bay. Republic of Korea: Suncheon; 2023.

[50] Bai J, Zhang H, Zhou H, Li S, Gao B, Chen P, et al. Winter coexistence in herbivorous waterbirds: Niche differentiation in a floodplain, Poyang Lake, China. Ecol Evol. 2021;11(23):16835–48. doi: 10.1002/ece3.8314 34938476 PMC8668764

[51] Powell LL, Ames EM, Wright JR, Matthiopoulos J, Marra PP. Interspecific competition between resident and wintering birds: experimental evidence and consequences of coexistence. Ecology. 2021;102(2):e03208. doi: 10.1002/ecy.3208 32981090

[52] Anne Bishop M, Feng-Shan L. Effects of farming practices in Tibet on wintering Black necked Crane ( Grus nigricollis ) diet and food availability. Biodiversity Science. 2002;10(4):393–8. doi: 10.17520/biods.2002054

[53] Cai T, Huettmann F, Guo Y. Using stochastic gradient boosting to infer stopover habitat selection and distribution of Hooded Cranes Grus monacha during spring migration in Lindian, Northeast China. PLoS One. 2014;9(2):e89913. doi: 10.1371/journal.pone.0089913 24587118 PMC3935961

[54] Davis CA, Smith LM. Foraging Strategies and Niche Dynamics of Coexisting Shorebirds at Stopover Sites in the Southern Great Plains. The Auk. 2001;118(2):484–95. doi: 10.1093/auk/118.2.484

[55] Ivlev VS. Experimental ecology of the feeding of fishes. New Haven, Connecticut, USA: Yale University Press; 1961.

[56] Wood KA, Stillman RA. Do birds of a feather flock together? Comparing habitat preferences of piscivorous waterbirds in a lowland river catchment. Hydrobiologia. 2014;738(1):87–95. doi: 10.1007/s10750-014-1921-6

[57] Kong D, Luo W, Liu Q, Li Z, Huan G, Zhang J, et al. Habitat use, preference, and utilization distribution of two crane species (Genus: Grus) in Huize National Nature Reserve, Yunnan-Guizhou Plateau, China. PeerJ. 2018;6:e5105. doi: 10.7717/peerj.5105 30042879 PMC6054782

[58] Bates D, Mächler M, Bolker B, Walker S. Fitting Linear Mixed-Effects Models Usinglme4. J Stat Soft. 2015;67(1). doi: 10.18637/jss.v067.i01

[59] Wang Y, Fan B, Ding Y, Pang S. The current situation and discussion on wetland ecological restoration of the middle and lower Yangtze River. China Water Research. 2011;13:4–6.

[60] Ribeiro PD, Iribarne OO, Navarro D, Jaureguy L. Environmental heterogeneity, spatial segregation of prey, and the utilization of southwest Atlantic mudflats by migratory shorebirds. Ibis. 2004;146(4):672–82. doi: 10.1111/j.1474-919x.2004.00301.x

[61] Gawlik DE. The effects of prey availability on the numerical response of wading birds. Ecological Monographs. 2002;72(3):329–46. doi: 10.1890/0012-9615(2002)072[0329:teopao]2.0.co;2

[62] Pearman PB, Guisan A, Broennimann O, Randin CF. Niche dynamics in space and time. Trends Ecol Evol. 2008;23(3):149–58. doi: 10.1016/j.tree.2007.11.005 18289716

[63] Newton I. Population limitation in birds: Academic press; 1998.

[64] Wan W, Zhou L, Song Y. Shifts in foraging behavior of wintering Hooded Cranes (Grus monacha) in three different habitats at Shengjin Lake, China. Avian Res. 2016;7(1). doi: 10.1186/s40657-016-0047-0

[65] Ma Z, Li B, Jing K, Zhao B, Tang S, Chen J. Effects of tidewater on the feeding ecology of hooded crane ( Grus monacha ) and conservation of their wintering habitats at Chongming Dongtan, China. Ecological Research. 2003;18(3):321–9. doi: 10.1046/j.1440-1703.2003.00557.x

[66] Gyimesi A, Franken MS, Feige N, Nolet BA. Human disturbance of Bewick’s Swans is reflected in giving‐up net energy intake rate, but not in giving‐up food density. Ibis. 2012;154(4):781–90. doi: 10.1111/j.1474-919x.2012.01253.x

[67] Cody ML. Competition and the structure of bird communities. Princeton University Press; 2020.

[68] O’connor RJ, Boaden PJS, Seed R. Niche breadth in Bryozoa as a test of competition theory. Nature. 1975;256(5515):307–9. doi: 10.1038/256307a0

[69] Park JY, Won PO. Wintering ecology of Bean goose (Anser fabalis) and Whitefronted goose (Anser albifrons) in Junam reservoirs. Korea Bulletin of Korea Institute of Ornithology. 1993;4:1–24.

[70] Ringelman JK. Waterfowl Management Handbook. Fish and Wildlife Leaflet. 1990;13(10):10.

[71] Witkowska M, Wesołowski W, Markiewicz M, Pakizer J, Neumann J, Ożarowska A, et al. The intensity of supplementary feeding in an urban environment impacts overwintering Mallards (Anas platyrhynchos) as wintering conditions get harsher. Avian Research. 2024;15:100205. doi: 10.1016/j.avrs.2024.100205

[72] Ohsako Y. Flock Organization, Dispersion and Territorial Behaviour of Wintering Hooded Cranes Grus monacha in Izumi and Akune, Kyushu. Japanese Journal of Ornithology. 1989;38(1):15–29. doi: 10.3838/jjo.38.15

[73] Robb GN, McDonald RA, Chamberlain DE, Bearhop S. Food for thought: supplementary feeding as a driver of ecological change in avian populations. Frontiers in Ecol & Environ. 2008;6(9):476–84. doi: 10.1890/060152

[74] Harris J, Mirande C. A global overview of cranes: status, threats and conservation priorities. Avian Research. 2023;4(3):189–209.

[75] Mi C, Møller AP, Guo Y. Annual spatio-temporal migration patterns of Hooded Cranes wintering in Izumi based on satellite tracking and their implications for conservation. Avian Res. 2018;9(1). doi: 10.1186/s40657-018-0114-9

[76] Jones JD, Kauffman MJ, Monteith KL, Scurlock BM, Albeke SE, Cross PC. Supplemental feeding alters migration of a temperate ungulate. Ecol Appl. 2014;24(7):1769–79. doi: 10.1890/13-2092.1 29210236

[77] Goss-Custard J. Role of winter food supplies in the population ecology of common British wading birds. Verhandlung Ornithologische Gesellschaft Bayern. 1981;23:125–46.

[78] Sutherland WJ. Predicting the consequences of habitat loss for migratory populations. Proceedings of the Royal Society of London Series B: Biological Sciences. 1996;263(1375):1325–7.

[79] Claassen R, Cattaneo A, Johansson R. Cost-effective design of agri-environmental payment programs: U.S. experience in theory and practice. Ecological Economics. 2008;65(4):737–52. doi: 10.1016/j.ecolecon.2007.07.032

[80] Dobbs TL, Pretty J. Case study of agri-environmental payments: The United Kingdom. Ecological Economics. 2008;65(4):765–75. doi: 10.1016/j.ecolecon.2007.07.030

[81] Végvári Z, Barta Z. Roost Site Selection of the Common Crane in Its Largest European Stop-Over Site. Ardea. 2015;103(2):175–81. doi: 10.5253/arde.v103i2.a6

